# Symptomatic carotid near-occlusion causes a high risk of recurrent ipsilateral ischemic stroke

**DOI:** 10.1007/s00415-019-09605-5

**Published:** 2019-11-07

**Authors:** Thomas Gu, Richard I. Aviv, Allan J. Fox, Elias Johansson

**Affiliations:** 1 Department of Clinical Science, Umeå University, Norrlands Universitetssjukhus, 907 37 Umeå, Sweden; 2 Department of Medical Imaging, Sunnybrook Health Science Center, University of Toronto, Toronto, Canada; 3 Emeritus, Department of Medical Imaging, Sunnybrook Health Science Center, University of Toronto, Toronto, Canada; 4 Wallenberg Center for Molecular Medicine (WCMM), Umeå University, Umeå, Sweden

**Keywords:** Stroke, Neurology, Carotid stenosis, Large vessel disease

## Abstract

**Objective:**

To assess the risk of recurrent ipsilateral ischemic stroke in patients with symptomatic near-occlusion with and without full collapse.

**Methods:**

Included were consecutive patients eligible for revascularization, grouped into symptomatic conventional ≥ 50% carotid stenosis (*n* = 266), near-occlusion without full collapse (*n* = 57) and near-occlusion with full collapse (*n* = 42). The risk of preoperative recurrent ipsilateral ischemic stroke was analyzed, or, for cases not revascularized within 90 days, 90-day risk was analyzed.

**Results:**

The risk of a preoperative recurrent ipsilateral ischemic stroke or ipsilateral retinal artery occlusion was 15% (95% CI 9–20%) for conventional ≥ 50% stenosis, 22% (95% CI 6–38%) among near-occlusion without full collapse and 30% (95% CI 16–44%) among near-occlusion with full collapse (*p* = 0.01, log rank test). In multivariate analysis, near-occlusion with full collapse had a higher risk of recurrent ipsilateral ischemic stroke (adjusted HR 2.6, 95% CI 1.3–5.3) and near-occlusion without full collapse tended to have a higher risk (adjusted HR 2.0, 95% CI 0.9–4.5) than conventional ≥ 50% stenosis. Only 24% of near-occlusion with full collapse underwent revascularization, common causes for abstaining were misdiagnosis as occlusion (31%), deemed surgically unfeasible (21%) and low perceived benefit (10%).

**Conclusions:**

Symptomatic carotid near-occlusion has a high short-term risk of recurrent ipsilateral ischemic stroke, especially near-occlusion with full collapse.

## Introduction

**Fig. 1:**
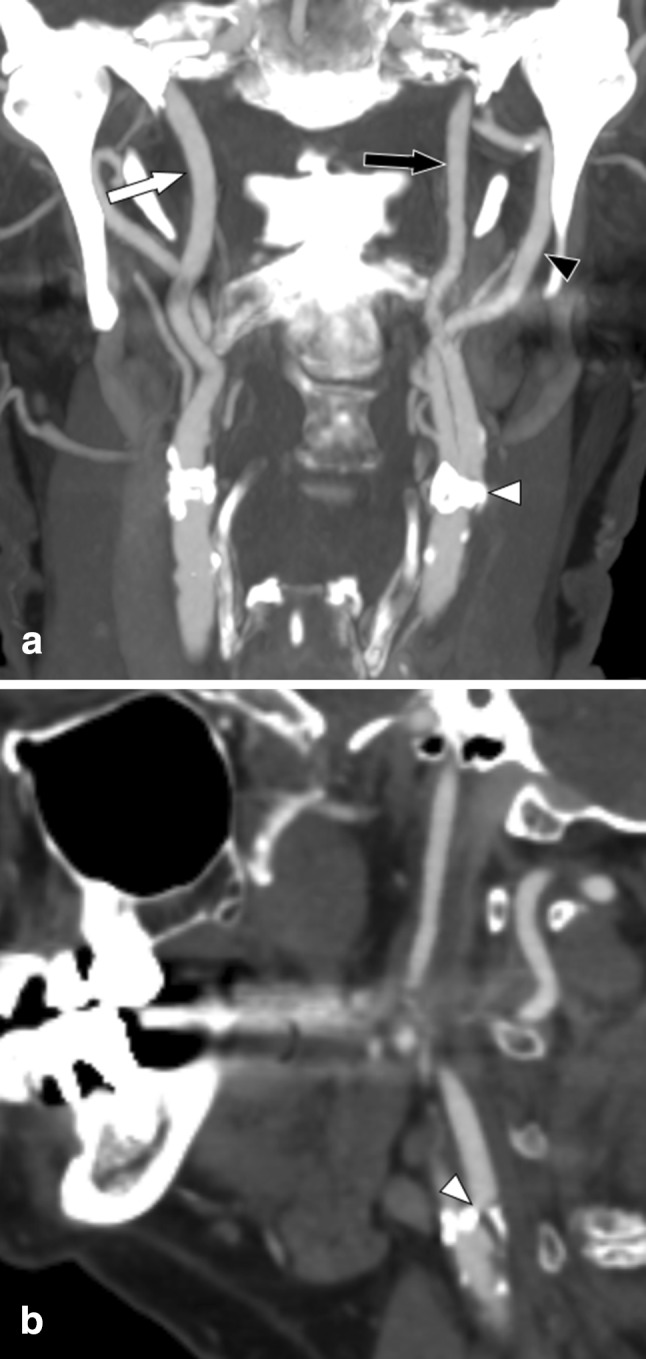
Left-sided symptomatic near-occlusion without full collapse; patient suffered a recurrent ipsilateral stroke 5 days after the exam. **a** Coronal view. Left distal ICA (black arrow, 3.3 mm) is smaller than right distal ICA (white arrow, 4.4 mm) and similar to left ECA (black arrowhead, 3.3 mm). Stenosis is hard to visualize (white arrowhead). **b** Sagittal view. Stenosis (white arrowhead) somewhat better visualized; lumen at stenosis is tight though but still difficult to assess. Axial source images (not displayed here) are usually most reliable to assess stenosis severity and with other features. A severe stenosis causing flow reduction was the most reasonable explanation of the small distal left ICA, interpreted as near-occlusion

**Fig. 2:**
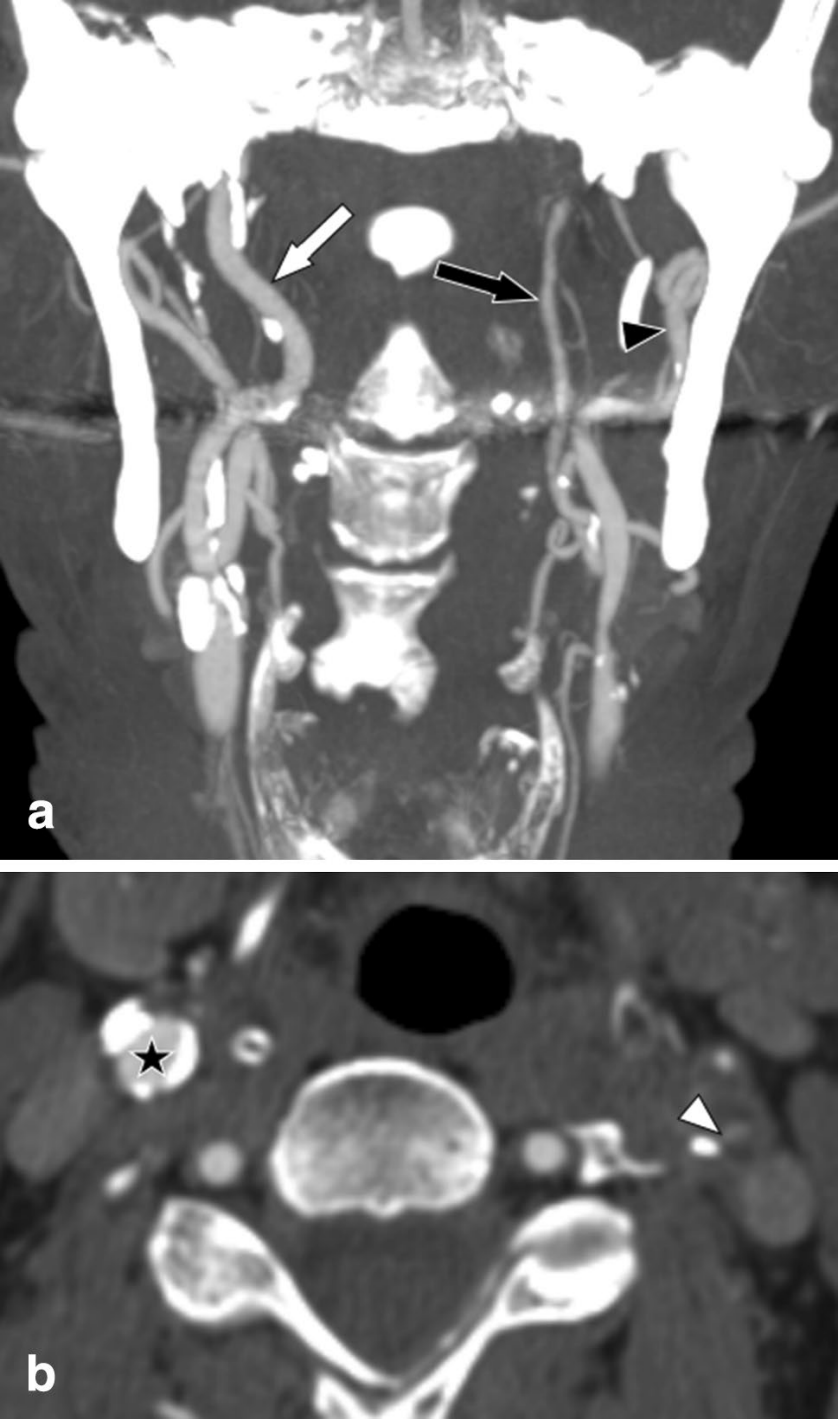
Left-sided symptomatic near-occlusion with full collapse; patient suffered a recurrent ipsilateral stroke 7 days after the exam. Left-sided symptomatic near-occlusion with full collapse. **a** Coronal view. Left distal ICA (black arrow, 1.2 mm) is clearly smaller than right distal ICA (white arrow, 3.7 mm) and smaller than left ECA (black arrowhead, 3.8 mm). Stenosis not seen in this projection, as the lumen was out-of-plane and so small on the image. **b** Axial view of the stenosis (white arrowhead, 0.8 mm). No relevant stenosis in left ICA (black start)

Carotid near-occlusion is a severe carotid stenosis that causes a reduction of the size of the internal carotid artery (ICA) distal to the stenosis [1–3]. The size reduction is likely a physiological response to flow reduction [2]. The distal size reduction is often moderate, with the distal artery being normal-appearing albeit small (near-occlusion without full collapse, Fig. 1), but is sometimes severe with a threadlike distal lumen (near-occlusion with full collapse, Fig. 2) [1–3].

In the pooled analysis of NASCET and ECST, 246 of 262 cases with near-occlusions were without full collapse and only 16 had criteria for near occlusions with full collapse [3]. These trials strongly suggested that carotid endarterectomy (CEA) is of less value for patients with near occlusion than for patients with severe stenosis but without near occlusion [3]. Accordingly, guidelines recommend best medical treatment, not revascularization, for patients with symptomatic near-occlusion [4, 5]. However, a recent consecutive study and a recent registry have found varying (5–43%) assessments of risk of recurrent ipsilateral ischemic stroke within 1 month of presenting event, and varying if near-occlusion with full collapse alone carries an increased risk [6, 7]. Since more than half of near-occlusions in NASCET and ECST were included > 4 weeks after last event, a high early risk might have been missed in these trials [3].

The purpose of this study was to analyze the short-term risk of ipsilateral ischemic stroke among a consecutive series of patients with symptomatic near-occlusion with and without full collapse diagnosed by CT angiography (CTA).

## Materials and methods

### Study population

We re-evaluated consecutive carotid CTAs performed or sent to the department of radiology at Norrland University Hospital during the period January 2010 to December 2014 (4440 CTA examinations from 4067 patients). Inclusion criteria were symptomatic ≥ 50% stenosis on CTA and being suitable candidates for evaluation for revascularization. Hence, exclusion criteria were severe co-morbidity (clearly causing patients to be unsuitable for secondary preventive revascularization, such as severe cognitive decline), major stroke as presenting event and > 6 months delay between presenting event and CTA; however, cases that did not undergo revascularization due to other causes, such as major recurrent stroke occurring after presentation and perceived poor risk/benefit after detailed evaluation were included.

### Clinical routine and medical records review

Umeå Stroke Center is the only center performing carotid interventions in northern Sweden, with 11 referring hospitals covering a population of 880,000. Two clinicians with stroke experience (TG, EJ) reviewed the medical records, blinded to the CTA findings except for side of stenosis. With standardized referral pathways from the 11 referring hospitals and universal use of digital medical records, access to information was excellent. As the preoperative evaluation routinely includes well-documented examinations by a specialist in neurology and internal medicine, data availability for events and co-morbidities was robust. Vascular baseline parameters, management, imaging findings and detailed descriptions of cerebrovascular events (previous and up to 90 days after presenting event) were collected. As clinical routine, post-operative assessments included neurological consult day 1–4, face-to-face stroke physician visit at day 30 or telephone with vascular surgery nurse in cases with missing 30-day data.

Patients were routinely prescribed antiplatelet therapy after the presenting event, but dual antiplatelet therapy with Clopidogrel was most often not given before CEA due to concerns of cervical hematoma. CEA in general anesthesia was the routine method. CEA indications were  ≥ 50% stenosis and recently (< 2 weeks) symptomatic and overall reasonable surgical candidates when considering cardiovascular risk profile, age (> 85 years rarely accepted), size of neurological deficit and other co-morbidities. Beyond 2 weeks after the last event, individual assessments were made with shorter delay, increasing age (> 65 years), severe conventional stenosis (≥ 70%) and male sex being markers for CEA indication. Based on preliminary local results [6], near-occlusion was considered to have roughly similar indication as a conventional stenosis. Angioplasty indications were high cardiovascular risk (*n* = 2, both awake procedures), previous CEA (*n* = 2) and for access when treating an early stroke recurrence with thrombectomy (*n* = 1).

The history and signs of all cerebrovascular events were critically assessed to adhere to our event definitions. Cases with multiple causes of stroke, such as concurrent atrial fibrillation, were only considered to have asymptomatic stenosis (and not analyzed) if there was sufficient evidence that the stenosis was clearly not the cause, such as clinical and/or radiological evidence of recent ischemia in one or more territories not supplied by the carotid artery. To adhere to conservative approach, occipital events were considered as posterior circulation events regardless of Circle of Willis status.

### CTA imaging

The CTAs were performed using several scanners and protocols reflecting standard of care imaging at each referring site. All cases were screened for eligibility by one reviewer (EJ) while all CTAs were reviewed by a second reviewer (AF, > 40 years of Neuroradiology experience), blinded to each other and to clinical information. Disagreements were resolved by consensus. Inter-rater reliability for near-occlusion was good: kappa 0.80, intra-rater reliability was kappa 0.75 and 0.88 for each observer. Near-occlusion was diagnosed when the carotid artery distal to the stenosis was judged to be reduced in size because of the stenosis. This was assessed using an interpretive approach weighing the information of four features (stenosis severity, distal ICA size, distal ICA asymmetry and ICA/ECA ratio) to find the best possible explanation for the findings [8]. A conservative approach was used, only calling near-occlusion when it was clearly the most reasonable diagnosis. One important mimic was always considered: Anatomical variance in distal ICA size caused by asymmetric Circle of Willis [9]: The ICA can be larger when it supplies posterior cerebral artery territory via a fetal origin in addition to middle cerebral artery, and larger still when that ICA supplies both anterior cerebral arteries via a large anterior communicating artery. The converse is smaller ICA when ICA supplies middle cerebral artery without much contribution to the posterior or anterior cerebral artery. Such anatomical variance of the ICA size can mimic near-occlusion mimic when coinciding with a stenosis [9] and were categorized as conventional stenosis.

Cases with contrast visible distal to the stenosis, but had not yet reached skull base at time of image capture were diagnosed as near-occlusion with full collapse, whereas occlusion was diagnosed when no contrast was seen beyond the lesion. Among the near-occlusions, full collapse was defined as a threadlike distal lumen, whereas those without full collapse had a normal-appearing albeit small distal ICA [1, 2]. Among cases with conventional stenosis, degree of stenosis was measured using established NASCET criteria [10].

### Definitions

The presenting event was the last event before seeking healthcare. The ipsilateral side was defined as the side of the presenting event. Stroke was defined according to the conservative 1976 WHOs definition [11]. TIA was defined as stroke, but lasting < 24 h. Retinal artery occlusion was defined as monocular vision loss with no apparent cause other than vascular origin lasting more than 24 h, often confirmed by retinal exam. Amaurosis fugax was defined as retinal artery occlusion, but lasting < 24 h and was often a clinical diagnosis without findings on retinal exam.

The primary endpoint was a preoperative recurrent ipsilateral ischemic stroke or ipsilateral retinal artery occlusion. Among cases that had not undergone revascularization within 90 days after presenting event, only events occurring within 90 days after presenting event were considered for the primary endpoint. Secondary endpoints were (1) ipsilateral ischemic stroke (2) any preoperative recurrent ipsilateral ischemic event (stroke, TIA, retinal artery occlusion or amaurosis fugax) and (3) any preoperative stroke. Safety endpoint was any stroke or death within 30 days after carotid revascularization.

### Statistical analysis

This was the pre-specified primary analysis of this cohort. We used mean, median, standard deviation (SD), intra-quartile range (IQR), 95% confidence intervals (95% CI), two-sided *χ*^2^-test, ANOVA, and Kruskal–Wallis test as appropriate. The primary endpoint and secondary endpoints of ipsilateral ischemic stroke and any preoperative ipsilateral ischemic event were analyzed with Kaplan–Meier curves with log rank test. The presenting event was index time; cases not reaching the endpoint were censored at carotid revascularization, death or 90 days. Bivariate and multivariate Cox regression was performed with the same index time and censoring as the Kaplan–Meier analysis, producing hazard ratios (HR). After bivariate analysis of all co-variates, a multivariate model was created using possible confounders, identified as associated (*p* < 0.1) with near-occlusion at baseline and/or with the primary outcome. Check for curve crossing, co-linearity and interaction revealed no relevant findings among included co-variates. Statistically significance was determined by *p* < 0.05. All analyses were performed in SPSS v24.0.

## Results

Of the screened 4067 patients, 642 had a ≥ 50% carotid stenosis, of which 379 were symptomatic. 19 patients were excluded due to severe co-morbidity (*n* = 6), major stroke as presenting event (*n* = 11) and > 6 months delay between presenting event and CTA (*n* = 2). Five cases had two distinctly separate symptomatic episodes and were therefore included twice. These were three conventional ≥ 50% stenosis and two near-occlusions (one with, one without full collapse); the two episodes were either > 6 months apart and contralateral (*n* = 2) or were > 1 year apart and ipsilateral, but did not undergo surgery for the first episode (*n* = 3). The final cohort therefore comprised of 365 incidents from 360 patients, all undergoing CTA within 6 months post presenting event (*n* = 362) or within 90 days before the presenting event and not repeated (*n* = 3).

Of included cases, 99 (27%; 95% CI 23–32%) had near-occlusion, remaining 266 conventional ≥ 50% stenosis. Of near-occlusions, 42 (42%; 95% CI 33–52%) were with full collapse, remaining 57 were without full collapse. Of conventional stenoses, 146 were 50–69%, 116 were ≥ 70%, and 4 were too calcified to categorize beyond being ≥ 50%. Cases with near-occlusion were younger and received fewer lipid lowering treatments at presentation than conventional ≥ 50% stenosis (Table 1). Near-occlusion without full collapse patients were less often hypertensive, but experienced more additional ipsilateral ischemic events within the 2 weeks before the presenting event than other groups. Revascularization treatment was performed in 225 (62%) cases: 219 CEA, 4 angioplasty with stenting, 1 angioplasty without stenting and 1 cervical by-pass. Cases with near-occlusion with full collapse underwent revascularization less often than cases with near-occlusion without full collapse or conventional ≥ 50% stenosis (*p* < 0.001, Table 1), rationale for not performing revascularization listed in Table 2. The 30-day risk of stroke or death after revascularization was 2.9% (5/171), 0% (0/43) and 20% (2/10) for conventional ≥ 50% stenosis, near-occlusion without full collapse and near-occlusion with full collapse respectively (*p* = 0.02).

**Table 1:** Comparisons of baseline factors, treatment when seeking health care, management and CTA measurements

	Conventional ≥ 50% stenosis (*n* = 266)	Near-occlusion without full collapse (*n* = 57)	Near-occlusion with full collapse (*n* = 42)	*p* value
Age mean (SD)	73 (8)	70 (10)	70 (8)	0.013*
Male sex *n* (%)	187 (70)	40 (70)	28 (67)	0.90^†^
Previous myocardial infarction *n* (%)	52 (20)	11 (19)	6 (14)	0.75^†^
Current angina *n* (%)	42 (16)	6 (11)	6 (14)	0.62^†^
Heart failure *n* (%)	20 (8)	3 (5)	2 (5)	0.67^†^
Current intermittent claudication *n* (%)	16 (6)	7 (12)	2 (5)	0.21^†^
Previous arterial Revascularization *n* (%)	52 (20)	9 (16)	8 (19)	0.82^†^
Atrial fibrillation *n* (%)	25 (9)	7 (12)	3 (7)	0.68^†^
Current smoking *n* (%)	41 (15)	15 (26)	8 (9)	0.15^†^
Diabetes *n* (%)	66 (25)	15 (26)	7 (17)	0.48^†^
Hypertension^a^*n* (%)	241 (91)	45 (79)	37 (88)	0.03^†^
Total Cholesterol mmol/l mean (SD)	4.9 (1.3)	5.3 (1.5)	4.9 (1.3)	0.26*
LDL Cholesterol mmol/l mean (SD)	2.9 (1.2)	3.3 (1.4)	3.0 (1.3)	0.17*
HDL Cholesterol mmol/l mean (SD)	1.26 (0.72)	1.09 (0.28)	1.29 (0.26)	0.21*
Medical treatment before seeking health care				
No AP/AC *n* (%)	123 (49)	27 (55)	28 (70)	0.05^†,^^#^
Single AP *n* (%)	90 (36)	17 (34)	8 (20)	0.14^†,^^#^
Dual AP *n* (%)	19 (8)	3 (6)	3 (8)	0.95^†,^^#^
AC *n* (%)	18 (7)	3 (6)	1 (3)	0.41^†,^^#^
Blood-pressure *n* (%)	197 (79)	34 (69)	27 (68)	0.14^†^
Lipid reducing *n* (%)	138 (55)	24 (49)	14 (34)	0.04^†^
Previous stroke *n* (%)	38 (14)	7 (12)	8 (19)	0.66^†^
Ipsilateral event < 14 days before presenting event *n* (%)	54 (20)	20 (35)	2 (5)	0.0012^†^
Presenting event: stroke *n* (%)	132 (50)	21 (37)	27 (64)	0.06^†^
Presenting event: TIA *n* (%)	97 (37)	23 (40)	12 (29)	
Presenting event: retinal artery occlusion *n* (%)	14 (5)	2 (4)	1 (2)	
Presenting event: amaurosis fugax *n* (%)	23 (9)	11 (19)	2 (5)	
Sought health care at other hospital *n* (%)	202 (76)	44 (77)	34 (81)	0.78^†^
Sought health care on the day of presenting event *n* (%)	201 (76)	38 (67)	33 (79)	0.31^†^
Days between presenting event and CTA median (IQR)	2 (0–4)	4 (1–14)	3 (0–9)	0.03^§^
Underwent revascularization *n* (%)	171 (64)	44 (77)	10 (24)	< 0.001^†^
Days between presenting event and revascularization median (IQR)	9 (6–15)	12 (6–24)	14 (9–24)	0.11^#^
Stenosis diameter mm mean (SD)	1.4 (0.5)	0.7 (0.2)	0.6 (0.3)	< 0.001*
Distal ICA diameter mm mean (SD)	4.3 (0.6)	2.9 (0.5)	1.1 (0.8)	< 0.001*
ICA ratio mean (SD)	0.99 (0.23)	0.64 (0.10)	0.30 (0.30)	< 0.001*
ICA/ECA ratio mean (SD)	1.65 (0.41)	1.09 (0.48)	0.38 (0.29)	< 0.001*
Percent NASCET-stenosis mean (SD)	68 (10)	Not applicable	Not applicable	–

*AC* anti-coagulant, *AP* anti-platelet

*ANOVA

^†^2-sided *χ*^2^-test

^#^The overall difference comparing regimens as a 4-group parameter with degrees of stenosis was *p* = 0.29, 2-sided *χ*^2^-test

^§^Kruskal–Wallis

^a^First available blood-pressure > 140/90 or use of blood-pressure medication when seeking health care

**Table 2:** Comparison of revascularization rate and causes for not performing revascularization

	Conventional ≥ 50% stenosis (*n* = 266)	Near-occlusion without full collapse (*n* = 57)	Near-occlusion with full collapse (*n* = 42)
Revascularization *n* (%)	171 (64)	44 (77)	10 (24)
Consent not possible *n* (%)	3 (1)	1 (2)	0 (0)
Interpreted as < 50% stenosis *n* (%)	42 (16)	1 (2)	1 (2)
Interpreted as occlusion *n* (%)	0 (0)	2 (4)	13 (31)
Asymptomatic progressed to occlusion *n* (%)	0 (0)	0 (0)	1 (2)
Symptomatic progression to occlusion *n* (%)	0 (0)	0 (0)	0 (0)
Clear mismanagement^a^*n* (%)	9 (3)	0 (0)	0 (0)
Perceived to have weak indication *n* (%)	11 (4)	1 (2)	4 (10)
Too high procedural risk due to co-morbidity *n* (%)	14 (5)	1 (2)	2 (5)
Patient refused *n* (%)	9 (3)	2 (4)	0 (0)
Technically unfeasible, cannot reach with clamp *n* (%)	1 (<1)	2 (4)	9 (21)
Technically unfeasible, other causes *n* (%)	2 (<1)	1 (2)	0 (0)
Major recurrent stroke *n* (%)	4 (2)	2 (4)	2 (5)

^a^Clear misinterpretation of cerebrovascular event or failure to notice imaging findings as only reasonable cause of not going ahead with revascularization

**Fig. 3:**
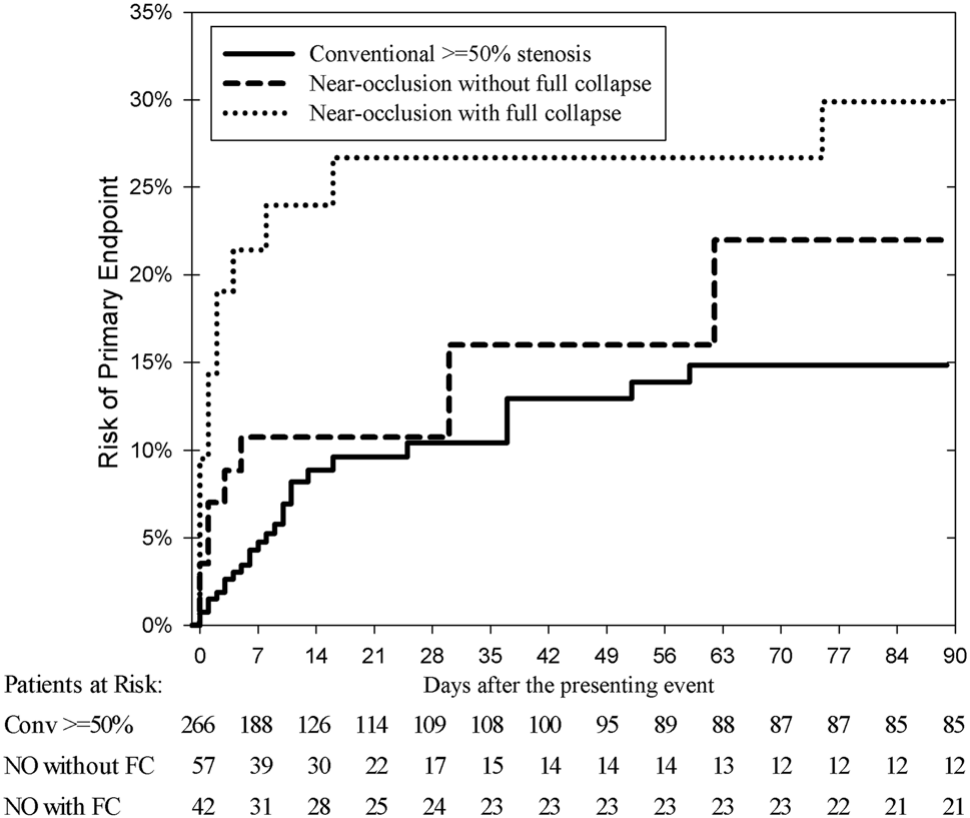
Kaplan–Meier analysis of the primary endpoint (recurrent ipsilateral ischemic stroke or retinal artery occlusion). Revascularization and death used as censors. Overall difference *p* = 0.012. Near-occlusion with full collapse compared to conventional ≥ 50% stenosis *p* = 0.003. Near-occlusion without compared to with full collapse *p* = 0.26. Near-occlusion without full collapse compared to conventional ≥ 50% stenosis *p* = 0.017. Abbreviations in patients at risk table: Conv ≥ 50% Sten: conventional ≥ 50% stenosis. NO with FC: near-occlusion with full collapse. NO without FC: near-occlusion without full collapse

All patients were followed until surgery (*n* = 214), death (*n* = 4) or 90 days (*n* = 147). During the 90-days follow-up period, a total of 46 patients reached the primary endpoint of a preoperative recurrent ipsilateral ischemic stroke or ipsilateral retinal artery occlusion (45 strokes, 1 retinal artery occlusion, all ipsilateral ischemic). There was only one clinically suspected retinal artery occlusion, confirmed with acute retinal exam. This occurred in 15% among conventional ≥ 50% stenosis, 22% among near-occlusion without full collapse and 30% among near-occlusion with full collapse, *p* = 0.012 (Fig. 3, Table 3). With conventional ≥ 50% stenosis as reference, the HR for the primary outcome was 1.5 (95% CI 0.7–3.4) for near-occlusion without full collapse and 2.7 (95% CI 1.4–5.4) for near-occlusion with full collapse (Table 4). After adjusting for confounders, near-occlusion with full collapse remained virtually unaffected (adjusted HR 2.6, 95% CI 1.3–5.3) while near-occlusion without full collapse approached a significant risk increase (adjusted HR 2.0, 95% CI 0.9–4.5).

**Table 3:** Risk of endpoints at five time points after presenting event

	All (*n* = 365)		Conventional ≥ 50% stenosis (*n* = 266)		Near-occlusion without full collapse (*n* = 57)		Near-occlusion with full collapse (*n* = 42)	
	*n*	% Risk (95% CI)	*n*	% Risk (95% CI)	*n*	% Risk (95% CI)	*n*	% Risk (95% CI)
Primary endpoint—ipsilateral ischemic stroke or retinal artery occlusion								
2 days	17	5 (3–7)	5	2 (0–3)	4	7 (0–14)	6	14 (4–25)
7 days	27	8 (5–10)	12	5 (2–7)	6	11 (3–19)	9	21 (9–34)
14 days	35	11 (7–14)	19	9 (5–13)	6	11 (3–19)	10	24 (11–37)
30 days	39	13 (9–17)	21	10 (6–15)	7	16 (3–29)	11	27 (13–40)
90 days	46	17 (13–22)	26	15 (9–20)	8	22 (6–38)	12	30 (16–44)
Secondary endpoint—ipsilateral ischemic stroke								
2 days	16	4 (2–7)	5	2 (0–3)	3	5 (0–11)	6	14 (4–25)
7 days	26	7 (5–10)	12	5 (2–7)	5	9 (2–16)	9	21 (9–34)
14 days	34	11 (7–14)	19	9 (5–13)	5	9 (2–16)	10	24 (11–37)
30 days	38	13 (9–17)	21	10 (6–15)	6	14 (2–26)	11	27 (13–40)
90 days	45	17 (12–22)	26	15 (9–20)	7	20 (4–35)	12	30 (16–44)
Secondary endpoint—ipsilateral ischemic stroke, TIA, retinal artery occlusion or amaurosis fugax								
2 days	49	13 (10–17)	33	12 (8–16)	6	11 (2–19)	10	24 (11–37)
7 days	71	20 (16–24)	48	19 (14–23)	10	18 (8–28)	13	31 (17–45)
14 days	82	24 (19–29)	54	22 (17–27)	14	28 (15–41)	14	34 (19–48)
30 days	87	27 (22–32)	57	24 (19–30)	15	33 (18–48)	15	36 (22–51)
90 days	96	32 (26–38)	64	30 (23–36)	16	38 (21–56)	16	39 (24–55)

Based on Kaplan–Meier estimates

**Table 4:** Bivariate and multivariate analysis of primary endpoint (recurrent ipsilateral ischemic stroke or retinal artery occlusion) with Cox regression

	Bivariate analysis		Multivariate analysis	
	HR (95% CI)	*p* value	HR (95% CI)	*p* value
Conventional ≥ 50% stenosis	1.0	Ref	1.0	Ref
Near-occlusion without full collapse	1.5 (0.7–3.4)	0.28	2.0 (0.9–4.5)	0.09
Near-occlusion with full collapse	2.7 (1.4–5.4)	0.005	2.6 (1.3–5.3)	0.006
Age (10-year increment)	1.1 (0.8–1.6)	0.49	1.2 (0.9–1.8)	0.26
Male sex	0.9 (0.5–1.6)	0.66	Not used	–
Previous myocardial infarction	1.1 (0.5–2.2)	0.78	Not used	–
Current angina	1.4 (0.7–2.8)	0.41	Not used	–
Heart failure	0.9 (0.3–2.8)	0.81	Not used	–
Current intermittent claudication	1.2 (0.4–3.4)	0.71	Not used	–
Previous arterial revascularization	1.4 (0.7–2.8)	0.28	Not used	–
Atrial fibrillation	1.8 (0.8–3.8)	0.14	Not used	–
Current smoking	0.8 (0.4–1.8)	0.64	Not used	–
Diabetes	0.7 (0.4–1.5)	0.41	Not used	–
Hypertension	2.6 (0.6–10.8)	0.18	2.6 (0.6–11.0)	0.19
Previous stroke	1.0 (0.5–2.3)	0.98	Not used	–
Ipsilateral event < 14 days before presenting event	0.7 (0.3–1.6)	0.41	0.8 (0.3–2.0)	0.69
Cerebral^a^ presenting event	7.8 (1.1–56.4)	0.04	7.0 (0.96–51.5)	0.06
Sought health care at other hospital	0.9 (0.5–1.8)	0.85	Not used	–

All co-variates with p < 0.1 in bivariate analysis or in baseline analysis used in the multivariate model

*HR* hazard ratio

^a^Stroke and TIA merged to “cerebral” event, compared to amaurosis fugax and retinal artery occlusion merged to “retinal” event: stroke and TIA had similar high risk of primary endpoint (*p* = 0.82), whereas only one case with retinal artery occlusion and none with amaurosis fugax reached the primary endpoint

Risk of ipsilateral ischemic stroke was very similar to primary outcome as only a single case of recurrent ipsilateral retinal artery occlusion differed (Table 3). Any ipsilateral event occurred in 96 patients, 90-day risk was 30% in conventional ≥ 50% stenosis, 38% in near-occlusion without full collapse and 39% in near-occlusion with full collapse, *p* = 0.29 (Table 3). There were four recurrent non-ipsilateral ischemic strokes: one thrombolysis associated hemorrhagic stroke, two posterior ischemic strokes and one contralateral ischemic stroke; all four had conventional ≥ 50% stenosis, no other ischemic recurrences and one patient had atrial fibrillation.

### Explorative analyses

Forty-four cases did not undergo revascularization as they were interpreted as < 50% by the clinician (Table 2). The 42 with conventional ≥ 50% stenosis in our re-assessment had mean 59%, SD 8% degree of stenosis and a similar 90-day risk of primary outcome (12%) as the remaining 224 cases with conventional ≥ 50% stenosis (16%), *p* = 0.53. Two near-occlusions were interpreted as < 50% stenosis as ultrasound had low flow velocity, but misinterpreted it as < 50% stenosis. Among conventional ≥ 50% stenoses, there was no association between the 90-day risk of the primary outcome and degree of stenosis, neither when assessed as continuous variable (HR 1.0, *p* = 0.99, Cox regression) or dichotomized into 50–69% (14%) and ≥ 70% (15%, *p* = 0.90, log rank). When limiting the analysis to the 140 cases not undergoing revascularization, 11 of 32 near-occlusions with full collapse suffered the primary outcome; 7, 14, and 90 day risks were 25%, 28% and 34% for near-occlusion with full collapse and with statistical difference in risk between the three stenosis groups (*p* = 0.035, log rank).

Comprehensive searches of local databases revealed that during the study period, 149 patients with symptomatic ≥ 50% stenosis were evaluated, but examined with ultrasound alone. Hence, 71% (365/514) of cases with symptomatic ≥ 50% stenosis during the study period were included as CTA was required for analysis.

## Discussion

The main finding of this study was that the risk of recurrent ipsilateral ischemic stroke was high among all patients with symptomatic near-occlusion, especially near-occlusion with full collapse. This confirms the high risk reported from a recent single-center study [6].

There are both similarities and differences between the current study and the pooled analysis of near-occlusions in NASCET and ECST [3]. Similar to NASCET and ECST, we used an interpretive approach of near-occlusion features for diagnosis, recognizing near-occlusion without full collapse and anatomical variant mimics [3]. Furthermore, a single expert interpreter and creator of the NASCET methodology (AF) interpreted all studies [1–3]. Our choice of endpoint was also similar to NASCET and ECST, as we used the time based definition of stroke (symptoms lasting > 24 h), not the novel ICD 11 stroke definition (which also includes imaging [12]), and that we included retinal artery occlusions in our primary endpoint [13]. However, we found that 42% of near-occlusions were with full collapse, whereas only 6% of near-occlusions in NASCET and ECST were with full collapse; [3] since the current study was a consecutive sample, this difference suggests a possible selection bias in these trials. Further, the current study found a high risk of recurrent stroke among all near-occlusions, whereas NASCET and ECST found a relatively low risk of recurrent stroke in the medical arm among all near-occlusions [3]. This was likely explained by difference in timing: The current study emphasized on first days after presenting event whereas these trials studied long-term risk, but often included near-occlusion cases with considerable delay after last event (51% > 4 weeks) [3].

As presented in a recent meta-analysis [14], cases with symptomatic near-occlusion that did not undergo revascularization had a relatively high risk of recurrent ischemic stroke. Two of the underlying studies had a similar approach as our current study, analyzing risk of early stroke recurrence in those with and without full collapse, but with differences in findings [6, 7]. We found a high risk among all near-occlusion and a possible risk difference between near-occlusion with and without full collapse. These recent studies reported a modest risk among near-occlusion [7] and low risk among near-occlusion without full collapse [6, 7]. These differences could be explained by registry methodology and imaging interpretation issues (near-occlusion diagnostics is difficult, near-occlusion without full collapse seems often overlooked) [1, 2, 6–10]. Current findings seems reliable because of a pre-specified hypothesis, relatively large size with number of near-occlusion types (nearly as large as both recent studies combined [6, 7]), consecutive assessment within a robust digital patient data environment with standardized referral pathways, standard of care imaging and well-documented specialist assessments.

A recent pooled analysis of three cohorts (totaling 377 cases) studying the risk of preoperative ipsilateral ischemic stroke within 90 days found that increasing age was a risk factor, cerebral presenting event tended (adjusted *p* = 0.06) to be a risk factor, but degree of stenosis was not (*p* = 0.66) [15]. We could not reproduce that older age caused increased risk of stroke, possibly because our near-occlusion cases were younger than the conventional ≥ 50% stenosis cases. We also found a strong trend that cerebral presenting events is a risk factor (again, adjusted *p* = 0.06). Near-occlusion could not be analyzed in the pooled cohort due to differences in diagnostics between the centers, but were merged into the ≥ 70% stenosis category [15]. In contrast, we demonstrate that degree of stenosis is an independent strong risk factor: near-occlusion with full collapse has a very high risk whereas near-occlusion without full collapse and all conventional ≥ 50% stenosis have a similar risk.

With the high risk of recurrent ipsilateral ischemic stroke in all near-occlusion and particularly in near-occlusion with full collapse, the possibility of treatment should be assessed in a randomized trial. However, some improvements seem reasonable before launching such a trial: Further work into prognosis is warranted to define optimal inclusion criteria. Many of the high-risk near-occlusion with full collapse cases were mistaken for occlusions in routine practice imaging (but correctly diagnosed by experts in retrospect), an issue likely not limited to our center, warranting improvements in diagnostics. Also, many of the high-risk near-occlusions were deemed technically unfeasible to treat and had a high risk of stroke when treated; similar risk has been suggested elsewhere [16]—pathophysiological work might reveal other treatment options than revascularization.

A limitation of this study is the non-randomized design. By virtue of the retrospective approach not all symptomatic stenosis cases during the study period underwent CTA, with 149 cases examined with ultrasound alone, the coverage was 71%. CTA was required in this analysis due to the poor sensitivity of carotid ultrasound as near-occlusion often have high flow velocity on ultrasound [17]; we noted 2 cases where typical near-occlusion findings on ultrasound (severe stenosis with low flow velocity in the stenosis) was misinterpreted as < 50% stenosis. However, the outcomes in the entire current cohort was very similar as a prior prospective cohort which did not require CTA use [18]; hence, selection bias in the current cohort does not seem to be a major concern. Cases undergoing revascularization might have had a different natural course than cases not treated, why late risk estimates (such as beyond 14 days after presenting event) are uncertain; however, few patients were treated within the first days after presenting event, making the main finding of early high risk more robust. Further, the possibility to assess the impact of medical therapy was limited as medical treatments were started with varying delay between cases at the time of highest risk, making causal relationships difficult to assess.

In summary, the short-term preoperative risk of recurrent ipsilateral ischemic stroke is high among all symptomatic near-occlusions, particularly near-occlusion with full collapse. As these findings question the current guideline recommendations of best medical treatment for symptomatic near-occlusion [4, 5], randomized controlled trials are warranted.

## Data Availability

The data that support the findings of this study are available from the corresponding author upon reasonable request.
